# The Role of Hematopoietic Cell Transplant in the Glycoprotein Diseases

**DOI:** 10.3390/cells9061411

**Published:** 2020-06-05

**Authors:** Brianna M. Naumchik, Ashish Gupta, Heather Flanagan-Steet, Richard A. Steet, Sara S. Cathey, Paul J. Orchard, Troy C. Lund

**Affiliations:** 1 Division of Pediatric Blood and Marrow Transplant, University of Minnesota, Minneapolis, MN 55455, USA; naumc003@umn.edu (B.M.N.); gupta461@umn.edu (A.G.); orcha001@umn.edu (P.J.O.); 2 Greenwood Genetic Center, Greenwood, SC 29646, USA; heatherfs@ggc.org (H.F.-S.); rsteet@ggc.org (R.A.S.); scathey@ggc.org (S.S.C.)

**Keywords:** glycoprotein disorders, lysosomal storage disease, hematopoietic cell transplant, enzyme replacement therapy, α-mannosidosis, aspartylglucosaminuria, β-mannosidosis, fucosidosis, galactosialidosis, sialidosis, mucolipidosis II, mucolipidosis III, Schindler Disease

## Abstract

The glycoprotein disorders are a group of lysosomal storage diseases (α-mannosidosis, aspartylglucosaminuria, β-mannosidosis, fucosidosis, galactosialidosis, sialidosis, mucolipidosis II, mucolipidosis III, and Schindler Disease) characterized by specific lysosomal enzyme defects and resultant buildup of undegraded glycoprotein substrates. This buildup causes a multitude of abnormalities in patients including skeletal dysplasia, inflammation, ocular abnormalities, liver and spleen enlargement, myoclonus, ataxia, psychomotor delay, and mild to severe neurodegeneration. Pharmacological treatment options exist through enzyme replacement therapy (ERT) for a few, but therapies for this group of disorders is largely lacking. Hematopoietic cell transplant (HCT) has been explored as a potential therapeutic option for many of these disorders, as HCT introduces functional enzyme-producing cells into the bone marrow and blood along with the engraftment of healthy donor cells in the central nervous system (presumably as brain macrophages or a type of microglial cell). The outcome of HCT varies widely by disease type. We report our institutional experience with HCT as well as a review of the literature to better understand HCT and outcomes for the glycoprotein disorders.

## 1. Introduction

The glycoproteinoses are a group of lysosomal storage diseases (alpha-mannosidosis, aspartylglucosaminuria, beta-mannosidosis, fucosidosis, galactosialidosis, sialidosis, mucolipidosis II, mucolipidosis III, and Schindler Disease) characterized by specific lysosomal enzyme defects and resultant buildup of undegraded glycoprotein substrates. This buildup causes a multitude of abnormalities in patients including skeletal dysplasia, inflammation, ocular abnormalities, liver and spleen enlargement, myoclonus, ataxia, psychomotor delay, and mild to severe neurodegeneration. Pharmacological treatment options exist through enzyme replacement therapy (ERT) for a few, but therapies for this group of disorders is largely lacking. Hematopoietic cell transplant (HCT) has been explored as a potential therapeutic option for many of these disorders, as HCT introduces functional enzyme-producing cells into the bone marrow and blood along with the engraftment of healthy donor cells in the central nervous system (presumably as brain macrophages or a type of microglial cell). The outcome of HCT varies widely by disease type. We report our institutional experience with HCT as well as a review of the literature to better understand HCT and outcomes for the glycoprotein disorders.

### 1.1. α-Mannosidosis 

α-Mannosidosis is an autosomal recessive disease caused by mutations in the *MAN2B1* gene on chromosome 19 that encodes the lysosomal enzyme α-mannosidase [1,2]. Historically, deficiency in α-mannosidase was classified as a mucopolysaccharidosis (Hurler-like syndrome) due to similar coarse facial features [3] but later was identified as a distinct entity. There is significant variability in clinical presentation as clinical phenotypes can range from mild to severe with no apparent genotype–phenotype correlation based on age at presentation and aggressive nature of the disease. Manifestations include skeletal abnormalities, namely scoliosis and sternum deformation; hearing impairment; immune deficiency; progressive developmental delays; and worsening intellectual disability [4]. Severe manifestations include progressive neurologic deterioration and is associated with myopathy. Elevated urinary presence of mannose-rich oligosaccharides accompanies α-mannosidosis. The diagnosis can be established by deficient α-mannosidase activity in leukocytes or fibroblasts and is confirmed by genetic testing for mutation in *MAN2B1*. Disease with earlier presentation is usually severe and commonly ends with early death [5,6,7]. Patients with milder phenotype often present in the second decade of life, have slower progression, and survive into adulthood with milder symptoms consisting of some hearing loss and intellectual disability.

### 1.2. HCT for α-Mannosidosis

Over 20 α-mannosidosis patients have undergone HCT, more than any of the other glycoprotein disorders. In a 2012 review, 17 patients who had undergone HCT were evaluated. Two patients in this study died shortly after transplant, yielding an 88% survival rate, which is similar to previously published survival rates for transplants in other nonmalignant diseases and similar to the reported 5-year survival rate after HCT in Hurler disease [8,9,10]. In the eight patients that were tested for peripheral blood α-mannosidase activity, the results were within the unaffected range. Developmentally, while all patients scored below average for their age group, they all made significant improvements after HCT and were able to participate in activities of daily living [10]. Additionally, there was no regression in previously learned skills. One patient who was 22 at the time of the study and had undergone HCT 12 years prior was able to live independently and have a normal social life. Hearing improvement was variable, and even though none achieved normal hearing capacity, some participants were able to discontinue hearing aids temporarily after transplant [10]. Skeletal abnormalities were more difficult to quantify, especially in the pediatric population, but some physicians reported stabilization or improvement [10]. 

Newer analyses specifically highlight the beneficial effects of HCT on central nervous system (CNS) pathology. For example, untreated α-mannosidosis patients have worsening white matter abnormalities, diminished myelination, and gliosis [11,12,13,14]. Additionally, cerebral magnetic resonance spectroscopy (MRS) has shown abnormal signals (presumed pathological) in two untreated patients that were not present in a patient who had undergone HCT [15]. In another case report, the authors identified a mannose-containing oligosaccharide resonance complex (MC) using MRS in a 2.5-year-old α-mannosidosis patient prior to HCT. Furthermore, they demonstrated complete disappearance of the MC at 9.5 months after HCT, which persisted even at 5.5 years post-HCT [12]. These reports suggest that MRS could be a useful modality to follow patients and to determine treatment effect. These studies also demonstrate that HCT can attenuate CNS disease, giving indirect evidence that healthy donor cells are playing a role in alleviating neuropathology.

HCT is considered a therapeutic option for α-mannosidosis and should be pursued as early as possible in order to minimize storage material accumulation and irreversible pathological changes, with the main concerns being neurologic function and skeletal development.

### 1.3. Enzyme Replacement Therapy (ERT) for α-Mannosidosis

Preclinical testing of recombinant human lysosomal acid α-mannosidase (rhLAMAN) in α-mannosidosis mouse models showed significant pathologic improvements including decline in substrate level and restoration of endogenous enzymatic levels in the CNS [16,17]. After 12 months of ERT, there was a marked decline of disease state biomarkers including urinary and serum oligosaccharides accompanied by improvement in behavioral, cognitive, and motor deficits [17]. A recent human trial of rhLAMAN in 33 patients demonstrated a reduction in serum oligosaccharide levels and clinical improvement in the 3-min stair climb test as well as improvements in endurance and pulmonary function [18]. Such clinical benefits can be linked to improved health-related quality of life [19]. Although there have been no head-to-head comparisons of the effectiveness of ERT and HCT in α-mannosidosis, ERT is not thought to have a significant blood–brain barrier penetration as stated by the European Medicines Agency, leaving HCT as the only modality with potential neurocognition preservation [20].

## 2. Aspartylglycosaminuria

Aspartylglycosaminuria (AGU) is recessively inherited with a relatively high prevalence in Finland due to the C163S, “Fin,” mutation in the *AGA* gene which is responsible for 98% of cases there [21]. AGU is characterized by insufficient aspartylglucosaminidase (AGA) activity that results in accumulation of aspartylglucosamine and other glycoaspargines throughout the body [21,22]. Often, the chief symptom is progressive developmental delay, but other clinical features include premature aging; coarse facial features in addition to other skeletal abnormalities; and an increased potential for arthritis, respiratory infections, psychiatric disorders, and epileptic seizures [21]. The typical course of disease includes slow development until the age of 13–16 years, a period of stability or slow decline until mid-20s, and rapid decline thereafter, with life expectancy estimated at 45–50 years. Cerebral pathology includes delayed myelination and poor differentiation between white and grey matter on T2-weighted images [23]. Currently, there is no curative treatment and the literature suggests treating associated symptoms according to the general therapeutic guidelines [21].

### 2.1. HCT for AGU

HCT was first performed on three AGU patients in the early 1990s, two of which achieved engraftment [23]. Three years after HCT, follow-up demonstrated disappearance of cellular “storage lysosomes” on rectal biopsy, increased distinction between cerebral white and grey matter, and heterozygous levels of AGA activity. A larger case series followed 19 patients with AGU, 5 of whom had undergone successful HCT including the two previously noted [24]. Though the sample size was limited, the findings of this study were concerning as those who underwent HCT faced more severe intellectual disabilities, achieving an average developmental age of 9 months inferior to the non-transplanted patients [24]. On the contrary, two patients (siblings) were followed further [25]. Five years after HCT, both patients made gains in temperament and attention and had not lost any learned abilities since transplant. Both patients also exhibited near normal cerebral spinal fluid of Tau-protein, a marker of neuronal and axonal degeneration/damage 5 years post-HCT [25]. MRI revealed an improvement of myelination in the youngest sibling and an arrest of demyelination in the older sibling [25]. This study indicated that there was a possible arrest in the disease course after HCT. At best, HCT may be AGU-disease modifying or arresting [26]. Due to these mixed results and the risk of serious complications with HCT, it is not currently the treatment of choice for patients diagnosed with AGU [21]. If HCT is pursued, donors should be unrelated or noncarrier relatives and the transplant should be done during infancy to spare as much intellectual function as possible.

### 2.2. ERT for AGU

Preclinical trials of ERT showed potential in AGU mouse models where in vitro administration of recombinant human AGA demonstrates correction of enzyme levels in AGA-deficient fibroblasts and lymphoblasts in addition to intracellular clearance of precursors [27]. After a two-week course of ERT, adult mouse models showed pathological correction in nonneural tissues, increased AGA activity in the brain to within 10% of normal levels, and reduced aspartylglucosamine stores by 20% [28]. In newborn mice, AGA treatment was even more effective, with clearance of 40% of the aspartylglucosamine accumulation in neural tissue [29]. Despite this preclinical data, no company is currently producing an ERT product for AGU.

## 3. Fucosidosis

Mutations in the *FUCA1* gene result in α-L-fucosidase deficiency resulting in fucosidosis—an ultrarare glycoprotein storage disorder with less than 100 reported cases [30,31]. Like the other glycoprotein storage disorders, there is great phenotypic variation, even within the families, with clinical features like coarse facial features, developmental delay, dysostosis multiplex, hepatosplenomegaly, seizures, and angiokeratoma [32,33]. A severe form of fucosidosis can present in infancy and frequently leads to death before age 10, while a milder form will present in early childhood but is associated with significant morbidity and mortality with death often before the age of 20 years.

### 3.1. HCT for Fucosidosis

Due to the rarity of the disease, only a few transplants for fucosidosis have been performed. The decision to do the very first HCT for this disease came following promising results in canines with fucosidase deficiency treated with HCT. These studies showed that HCT normalized alpha-L-fucosidase enzyme activity in leukocytes, plasma, and neural and visceral tissues [34,35]. HCT performed prior to clinical findings delayed the onset and reduced the severity of disease, while HCT performed after symptom onset was not effective [35]. Transplantation was first pursued in humans when an asymptomatic 7-month-old child was diagnosed with fucosidosis after the diagnosis of the symptomatic older brother [36]. Prior to transplant, MRI imaging demonstrated hyperintense cerebral and cerebellar white matter, indicating delayed myelination. Twenty days after HCT, the patient achieved increasing but subnormal levels of plasma α-fucosidase and normal levels in leukocytes, while urine analysis demonstrated near total disappearance of fucosidosis-associated oligosaccharides. One year after HCT showed a social, happy child with low-average range of abilities including locomotor, speech, and hearing delays. Cerebral spinal fluid (CSF) analysis one year after transplant demonstrated that the donor-derived enzyme had reached the CNS. Cerebral imaging demonstrated improved myelination in the cerebral hemispheres, internal capsule, and basal ganglia. The patient’s clinical disease course appeared to be markedly better than the untransplanted sibling. This first HCT showed signs that transplant could be a potential therapy option for children diagnosed with fucosidosis if performed before clinical onset.

A similar outcome was found in a minimally symptomatic child transplanted at 11 months of age after an older sibling was diagnosed with fucosidosis [33]. At four years post-HCT, there were major improvements in MRI findings, psychomotor development, swallowing, and number of pulmonary infections. The disease course was altered in comparison to the older sibling who progressed to death at age 7. Even with early transplant, the patient still exhibited delays in coordination and sociability but did gain the ability to walk alone at the age of 30 months [33]. Another case report summarized an umbilical cord blood (UCB) HCT in a symptomatic 3-year-old girl, noting that no further neurological deterioration after HCT was detected, cranial MRI results at two years post-HCT improved, MRS showed a prior lactic acid peak disappearing, and dysostosis multiplex persisted [32]. Three other patients underwent HCT for this disease; two of which showed slowing of neurologic progression and a dramatic reduction in respiratory infections (a common improvement after HCT for LSD) [37]. In consideration of these few case reports and despite clinical variability, HCT could be beneficial when it is performed early before significant symptoms are present.

### 3.2. ERT for Fucosidosis

ERT is still in preclinical development for fucosidosis. Interestingly, it is the first glycoprotein disorder to use intracisternal ERT to deliver a recombinant enzyme directly to the CNS in a canine model [38]. It was found that the enzyme reached all areas of the CNS but were higher near the injection site (39%–73% of normal) and lower in deep brain structures (2.6%–5.5% of normal). Ultimately, the study showed that direct CNS delivery of enzyme was safe; however, therapeutic levels were not achieved in “far reaching areas” as substrate levels did not decline substantially in those regions. A follow-up study (in the same experiment) showed partial improvement in neuropathology following intracisternal ERT, suggesting that perhaps a more intensive ERT protocol could improve outcomes [38].

## 4. Sialidosis

Mutations in *NEU1* lead to deficiency in lysosomal neuraminidase (NEU1) and results in the glycoproteinosis termed sialidosis due to the accumulation of sialyated glycoconjugates [39]. Two phenotypes exist. Type I is a mild, non-neuropathic (normosomatic) form that presents in the second decade of life and is associated with ataxia, myoclonus, and ocular defects [40]. Type II sialidosis is a severe, neuropathic presentation with much earlier onset. Clinical manifestations of type II sialidosis consist of mucopolysaccharidosis-like features including myoclonus, coarse facies, dysostosis multiplex, progressive intellectual delay, and sometimes the presence of a cherry-red spot. For unclear reasons, a subset of patients with type II sialidosis develop nephropathy due to abnormal kidney storage material accumulation [39,41].

### 4.1. HCT in Sialidosis

The first HCT occurred with a patient that presented at 1.5 months of age with failure to thrive, dysostosis multiplex, and dysmorphic features [39]. Urine and protein analyses led to the diagnosis of sialidosis prior to the onset of clear neurological impairment. Allogenic HCT was performed at 9 months of age with successful engraftment. Asymptomatic proteinuria was noted at 25 months of age, and a subsequent renal biopsy showed large cytoplasmic vacuolation resulting in enlargement of glomerular epithelial and tubular cells. The patient went on to develop renal failure, leading to hemodialysis at age 6, and subsequently underwent renal transplant a year later. Despite the absence of major complications, by age 11, the patient remained in poor condition with significant psychomotor retardation and musculoskeletal involvement. While HCT prevented the rapid and severe encephalopathy associated with type II sialidosis, HCT did not prevent renal disease or failure. It is unclear as to why HCT did not prevent kidney disease, but it is worth noting that HCT fails to prevent renal pathology in other lysosomal storage disorders such as has been observed in the Fabry’s disease mouse model [42]. Due to failure to attenuate nephropathy and psychomotor retardation, the effect of HCT in type II sialidosis is still an open question.

### 4.2. ERT for Sialidosis

Preclinical experiments in mouse models indicate that ERT can attenuate nephropathy, restore Neu1 activity in multiple tissues, and reduce lysosomal storage [43]. Limitation to ERT includes inefficient crossing of rNeu1 into the blood–brain barrier. This was confirmed in studies showing that neuraminidase activity was restored in all tissues except the brain and ears after ERT [43]. Also, it was also described that ERT triggered severe immune response against the exogenous enzyme, having implications for future use in sialidosis patients [43].

## 5. β-Mannosidosis

β-mannosidosis is an autosomal recessive disorder caused by mutations in *MANBA* resulting in the lack of the β-mannosidase enzyme and accumulation of oligosaccharides in lysosomes [44]. Clinical presentation is quite variable, even when accompanied by null mutations but may include facial dysmorphism, neurocognitive deficits (or delays), angiokeratomas, hearing loss, and aggressive/hyperactive behavior [45].

### 5.1. HCT in β-mannosidosis

Only one known HCT has occurred in a patient with β-mannosidosis [46]. HCT was performed for a 4-year-old boy who was diagnosed after presenting with macrocephaly and gross motor delays including deficits in balance and gait. The patient achieved engraftment, near normal CSF and plasma enzyme levels, and displayed a dramatic drop in urine oligosaccharides following HCT. At 2 years post-HCT, the patient had persistent impairment in motor function and a decline in visual/motor integration, memory, and overall IQ [46]. The limited experience with HCT in this disease suggests that the peripheral biochemical defect can be corrected with successful donor engraftment, but the extent of overall musculoskeletal and neurocognitive benefits have yet to be fully realized.

### 5.2. ERT for β Mannosidosis

There are no ongoing preclinical studies or clinical trials for ERT in β-mannosidosis.

## 6. Mucolipidosis Type II

Mucolipidosis type II (MLII) is an autosomal recessive glycoprotein disorder resulting from mutation in the *GNPTAB* gene and loss of N-acetylglucosamine (GlcNAc)-1-phosphotransferase activity [47]. Functional *GNPTAB* is present in the Golgi and transfers phosphate moieties to the N-glycans on select lysosomal enzymes that are destined to be trafficked to the lysosome. Aberrant GlcNAc-1-phosphotransferase function results in lysosomal enzymes being mis-targeted to the extracellular compartment, where they contribute to the pathophysiology of disease (in addition to the loss of their native lysosomal function) [48,49]. On phase-contrast microscopy, dense granules filling the cytoplasm define the pathologic “inclusion cell”, which led to the original name of “I-cell Disease” for MLII. Null *GNPTAB* mutations result in a severe storage disease that is often diagnosed in infancy or prenatally. Death due to cardio-pulmonary disease is common in the first decade, and early clinical findings include dysostosis multiplex, growth failure, marked developmental delay, and generalized hypotonia [50,51,52].

### 6.1. HCT for MLII

Lund et al. summarized the outcomes of 22 MLII patients who underwent allogenic HCT [47]. Patients were transplanted at a median age of 9 months; patients were 3 months after diagnosis (median). Fourteen patients had UCB transplants, with the remaining receiving bone marrow from related or unrelated donors. Nineteen patients achieved primary engraftment, while one patient underwent two additional transplants after graft failures. Only 6 patients remained alive at last follow-up, with a median time to death of 27.6 months. After HCT, most caregivers reported that patients required supportive measures such as gastrostomy tubes and ventilator support along with outcomes such as impaired ambulation and intellectual delay. Only two patients survived past the age of 10 years, with one ultimately being determined to have attenuated MLII, an intermediate form of the disease (not known at the time of HCT). It is not clear whether HCT improved neurocognitive development.

Even after transplant, patients continued to exhibit extracellular secretion of many lysosomal enzymes, resulting in very high plasma levels perhaps contributing to lack of efficacy of HCT in this disease. Furthermore, there was little change in overall survival for MLII patients after HCT compared to historic reports [47]. Therefore, HCT seems to be inefficient at significantly affecting the MLII disease process even when HCT is performed in a young age. This may be due to the severity of disease or other factors such as continued inappropriate extracellular secretion posttransplant from non-hematopoietic cell types. HCT would not inhibit the extracellular action of these mis-targeted enzymes and, therefore, would not rescue pathology. Nonetheless, more effective therapies are in need for development.

### 6.2. ERT for MLII

There are no ongoing preclinical studies or clinical trials for ERT for MLII. ERT for this condition is a challenging prospect in light of the multiple hydrolases that are deficient in MLII lysosomes. An understanding of how loss of specific hydrolases impacts affected tissues is poorly understood, and there is growing evidence that some of the pathology may be caused by hypersecretion of enzymes into the extracellular space, a molecular consequence of MLII that would not be addressed by ERT [48,49].

## 7. Mucolipidosis Type III

Mucolipidosis type III (MLIII) is a rare autosomal recessive disorder with reduced GlcNAc-1-phosphotransferase activity as with MLII [53]. MLIII can be broken into two subtypes: alpha/beta or gamma, depending on the defective subunit of the enzyme [54,55]. A recent review of 13 adult patients demonstrated variability in clinical presentation [56]. Four of these patients were originally misdiagnosed with a type of mucopolysaccharidosis. Skeletal abnormalities were the primary impairment, with all 13 displaying dysostosis multiplex and secondary osteoarthritis. Surgical interventions due to destruction of cartilage and bone lesions are common in the second or third decades of life, especially total hip replacements. Importantly, most patients experience normal cognitive function or only mild cognitive impairment. Despite some severely affected patients that may die in childhood, most survive into adulthood suffering primarily from the progressive skeletal problems, joint destruction, bone pain, and cardiopulmonary dysfunction. Currently, there is no curative treatment, and HCT has not been pursued because the risks of HCT do not seem to outweigh the relatively attenuated disease course of MLIII (compared to MLII). Secondarily, as with all other LSDs, the dysostosis multiplex would not likely be affected even with “successful” HCT.

### ERT for MLIII

There are no ongoing clinical trials or preclinical work with ERT for MLIII.

## 8. Galactosialidosis

The lysosomal disorder galactosialidosis (GS) results from a mutation in the CTSA gene and from deficiency in the lysosomal carboxypeptidase protective protein/cathepsin A (PPCA) [57], leading to insufficient β-galactosidase (β-Gal) and N-acetyl-α-neuraminidase (NEU1) activities [58]. This deficit leads to a buildup of sialyloligosaccharides and glycopeptides. Like sialidosis, sialyloligosacchariduria is present in this disorder. Manifestations of GS include coarse facies, a cherry-red spot, and foamy bone marrow cells [59,60]. There is also a severe neurological component that is marked by ataxia, diffuse leukoencephalopathy, and severe cognitive impairment. GS can be subdivided into early infantile, late infantile, and juveniles/adult forms demonstrating the spectrum of severity [61]. Many of those diagnosed with early infantile type do not survive past their first year of life, while those diagnosed with the late infantile form can survive well into adulthood. The juvenile/adult type presents any time after infancy and can have a more severe presentation than the late infantile type due to severe neurologic impairment (myoclonus, cerebellar ataxia, generalized seizures, and progressive cognitive impairment) [61].

### 8.1. HCT for Galactosialidosis

HCT with donor bone marrow cells that overexpress *PPCA* adoptively transferred into the *PPCA*^-/-^ mouse model corrects the systemic pathology of galactosialidosis and partially corrects the CNS pathology [62,63]. Other studies have retrovirally introduced a normal copy of *PPCA* into *PPCA*^-/-^ hematopoietic progenitor cells [64]. Transplantation of these gene-corrected cells led to resolution of systemic pathology with a stronger CNS response and correction of coordination defects in *PPCA^-/-^* mice [64]. These studies show that HCT or gene therapy could be a promising option for patients diagnosed earlier in the disease course, but thus far, use of HCT in these patients has not been reported.

### 8.2. ERT for Galactosialidosis

The baculovirus-insect cell expression system has been utilized to generate recombinant human PPCA [65]. Infusion of PPCA or combination PPCA and NEU1 into 1-month-old *PPCA^-/-^* mice completely corrected the GS phenotype, and normal cathepsin A activity was demonstrated two weeks after ERT was initiated. These preclinical findings provide a positive prelude for future clinical trials in ERT.

## 9. Schindler Disease

Schindler disease is an autosomal recessive disorder resulting from mutations in the *NAGA* gene and reduced α-N-acetylgalactosaminidase (α-NAGA) deficiency [66,67]. Like the other LSDs, there is a spectrum of clinical severity that is genotype dependent [68]. Those with the infantile type (type I) may appear healthy until about one year of age when they begin to lose previously acquired developmental milestones [69]. This condition is neurologically progressive, leading to seizures and death during childhood. A milder form of α-NAGA deficiency known as type II or Kanzaki disease presents with less severe symptoms such as mild cognitive impairment, sensorineural hearing loss, and angiokeratomas [70]. An intermediate form also exists (type III).

### HCT for Schindler Disease

Given the rarity of the disorder, HCT has not yet been pursued as a therapeutic option, but it is possible that, given the LSD-related pathophysiology, HCT could be used to attenuate this disorder and to alter the progressive natural history.

## 10. Discussion

Many of the glycoproteinoses described here lead to devastating physical and intellectual complications and often premature death. Insufficient enzymatic activity due to genetic defects results in intralysosomal substrate accumulation and subsequent pathophysiology. The foundation for the role of HCT in lysosomal storage disease lies with observation by Neufeld et al. that cells defective in lysosome enzyme production can take up mannose 6-phosphate-bearing enzyme from neighboring healthy cells, thereby “cross-correcting” the defect [71,72]. This observation was fully realized clinically when HCT proved to attenuate some of the clinical and biochemical features of mucopolysaccharidosis type I-H (Hurler syndrome) and type VI (Maroteaux-Lamy syndrome) [73,74]. Clinicians theorized that HCT could be extrapolated to a wide variety of LSDs including the glycoproteinoses with the published outcomes as we have summarized. Though HCT and ERT are efficacious in treating many of the visceral manifestations of these storage diseases, peripheral delivery of ERT does not cross the blood–brain barrier efficiently, leaving neurological manifestations of LSD unmodified. Hematopoietic cells can cross the blood–brain barrier (potentially differentiating in microglia or microglia-like cells), distributing the functional enzyme to neighboring deficient glia and neurons, thereby allowing a positive impact on the CNS disease pathophysiology [75,76,77,78]. While it is not completely understood, not all LSDs respond to HCT equally as outlined above. This is likely due to the vast differences in genetic cause, disease mechanism, and disease severity. However, for those that do, HCT can attenuate neurocognitive loss, can preserve quality of life, and can prolong survival for patients if performed early. Further studies are needed to explore how efficiently enzymes that are derived from transplanted hematopoietic cells are modified with mannose 6-phosphate residues, as this may represent an important variable in their ability to be taken up from the bloodstream and to cross-correct the affected tissues. Differences in the processing of HCT-derived enzymes might also explain the variable therapeutic benefit seen for some conditions when comparing HCT and ERT.

## 11. Conclusions

Some glycoproteinoses respond well to HCT when pursued early, as outlined in Table 1. The majority of the experience is with α-mannosidosis, aspartylglycosaminuria, fucosidosis, and MLII, with results ranging from positive to mixed and to poor. ERT is being studied for several of these ultrarare diseases with positive results in animal models, but clinical translation has often been slow. Importantly, HCT can be considered for some glycoproteinoses, especially if the affected individual is diagnosed very early in life. Given the rarity of these diseases, patients and families should be evaluated on a case by case basis in consultation with physicians and centers having transplant experience in storage diseases.

## Figures and Tables

**Table 1:** Summary of glycoprotein disorders and outcomes with Hematopoietic cell transplant (HCT) or enzyme replacement therapy (ERT).

Glycoprotein Disorder	HCT	ERT
α-Mannosidosis	Positive outcomes when pursued early	Positive preclinical results (mouse)
Aspartylglycosaminuria	Mixed results but possible attenuation when pursued early	Positive preclinical results (mouse)
Fucosidosis	Positive outcomes when performed before symptom onset	Positive preclinical results (canine)
Sialidosis	Prevents encephalopathy but not renal failure or psychomotor impairment	Prevents nephropathy in mouse models, NEU1 not restored in brain
β-Mannosidosis	One known case with stable outcome	No known studies
Mucolipidosis II	Likely does not improve outcomes	No known studies
Mucolipidosis III	No known cases	No known studies
Galactosialidosis	No known cases but positive mouse studies	Positive preclinical results (mouse)
Schindler’s Disease	No known cases	No known studies

## References

[1] Riise H.M., Berg T., Nilssen O., Romeo G., Tollersrud O.K., Ceccherini I. (1997). Genomic structure of the human lysosomal alpha-mannosidase gene (MANB). Genomics.

[2] Wakamatsu N., Gotoda Y., Saito S., Kawai H. (1997). Characterization of the human MANB gene encoding lysosomal alpha-D-mannosidase. Gene.

[3] Öckerman P. (1967). Generalised Storage Disorder Resembling Hurlers Syndrome. Lancet.

[4] Malm D., Nilssen Ø. (2008). Alpha-mannosidosis. Orphanet. J. Rare Dis..

[5] Bach G., Kohn G., Lasch E.E., El Massri M., Ornoy A., Sekeles E., Legum C., Cohen M.M. (1978). A new variant of mannosidosis with increased residual enzymatic activity and mild clinical manifestation. Pediatr. Res..

[6] Desnick R.J., Sharp H.L., Grabowski G.A., Brunning R.D., Quie P.G., Sung J.H., Gorlin R.J., Ikonne J.U. (1976). Mannosidosis: Clinical, morphologic, immunologic, and biochemical studies. Pediatr. Res..

[7] Autio S., Louhimo T., Helenius M. (1982). The clinical course of mannosidosis. Ann. Clin. Res..

[8] Miano M., Labopin M., Hartmann O., Angelucci E., Cornish J., Gluckman E., Locatelli F., Fischer A., Egeler R.M., Or R. (2007). Haematopoietic stem cell transplantation trends in children over the last three decades: A survey by the paediatric diseases working party of the European Group for Blood and Marrow Transplantation. Bone Marrow Transpl..

[9] Staba S.L., Escolar M.L., Poe M., Kim Y., Martin P.L., Szabolcs P., Allison-Thacker J., Wood S., Wenger D.A., Rubinstein P. (2004). Cord-blood transplants from unrelated donors in patients with Hurler’s syndrome. N. Engl J. Med..

[10] Mynarek M., Tolar J., Albert M.H., Escolar M.L., Boelens J.J., Cowan M.J., Finnegan N., Glomstein A., Jacobsohn D.A., Kuhl J.S. (2012). Allogeneic hematopoietic SCT for alpha-mannosidosis: An analysis of 17 patients. Bone Marrow Transplant..

[11] Borgwardt L., Lund A.M., Dali C.I. (2014). Alpha-mannosidosis—a review of genetic, clinical findings and options of treatment. Pediatric Endocrinol. Rev. Per..

[12] Danielsen E.R., Lund A.M., Thomsen C. (2013). Cerebral Magnetic Resonance Spectroscopy Demonstrates Long-Term Effect of Bone Marrow Transplantation in alpha-Mannosidosis. Jimd Rep..

[13] Dietemann J.L., Filippi de la Palavesa M.M., Tranchant C., Kastler B. (1990). MR findings in mannosidosis. Neuroradiology.

[14] Gutschalk A., Harting I., Cantz M., Springer C., Rohrschneider K., Meinck H.M. (2004). Adult alpha-mannosidosis: Clinical progression in the absence of demyelination. Neurology.

[15] Avenarius D.F., Svendsen J.S., Malm D. (2011). Proton nuclear magnetic resonance spectroscopic detection of oligomannosidic n glycans in alpha-mannosidosis: A method of monitoring treatment. J. Inherit. Metab. Dis..

[16] Stroobants S., Damme M., Van der Jeugd A., Vermaercke B., Andersson C., Fogh J., Saftig P., Blanz J., D’Hooge R. (2017). Long-term enzyme replacement therapy improves neurocognitive functioning and hippocampal synaptic plasticity in immune-tolerant alpha-mannosidosis mice. Neurobiol. Dis..

[17] Damme M., Stroobants S., Lüdemann M., Rothaug M., Lüllmann-Rauch R., Beck H.C., Ericsson A., Andersson C., Fogh J., D’Hooge R. (2015). Chronic enzyme replacement therapy ameliorates neuropathology in alpha-mannosidosis mice. Ann. Clin. Transl. Neurol..

[18] Adam J., Malone R., Lloyd S., Lee J., Hendriksz C.J., Ramaswami U. (2019). Disease progression of alpha-mannosidosis and impact on patients and carers—A UK natural history survey. Mol. Genet. Metab. Rep..

[19] Lund A.M., Borgwardt L., Cattaneo F., Ardigo D., Geraci S., Gil-Campos M., De Meirleir L., Laroche C., Dolhem P., Cole D. (2018). Comprehensive long-term efficacy and safety of recombinant human alpha-mannosidase (velmanase alfa) treatment in patients with alpha-mannosidosis. J. Inherit. Metab. Dis..

[20] Ceccarini M.R., Codini M., Conte C., Patria F., Cataldi S., Bertelli M., Albi E., Beccari T. (2018). Alpha-Mannosidosis: Therapeutic Strategies. Int. J. Mol. Sci..

[21] Arvio M., Mononen I. (2016). Aspartylglycosaminuria: A review. Orphanet J. Rare Dis..

[22] Mononen I., Fisher K.J., Kaartinen V., Aronson N.N. (1993). Aspartylglycosaminuria: Protein chemistry and molecular biology of the most common lysosomal storage disorder of glycoprotein degradation. FASEB J..

[23] Autti T., Santavuori P., Raininko R., Renlund M., Rapola J., Saarinen-Pihkala U. (1997). Bone-marrow transplantation in aspartylglucosaminuria. Lancet.

[24] Arvio M., Sauna-Aho O., Peippo M. (2001). Bone marrow transplantation for aspartylglucosaminuria: Follow-up study of transplanted and non-transplanted patients. J. Pediatrics.

[25] Malm G., Mansson J.E., Winiarski J., Mosskin M., Ringden O. (2004). Five-year follow-up of two siblings with aspartylglucosaminuria undergoing allogeneic stem-cell transplantation from unrelated donors. Transplantation.

[26] Ringden O., Remberger M., Svahn B.M., Barkholt L., Mattsson J., Aschan J., Le Blanc K., Gustafsson B., Hassan Z., Omazic B. (2006). Allogeneic hematopoietic stem cell transplantation for inherited disorders: Experience in a single center. Transplantation.

[27] Mononen I., Heisterkamp N., Dunder U., Romppanen E.L., Noronkoski T., Kuronen I., Groffen J. (1995). Recombinant glycosylasparaginase and in vitro correction of aspartylglycosaminuria. Faseb. J..

[28] Dunder U., Kaartinen V., Valtonen P., Vaananen E., Kosma V.M., Heisterkamp N., Groffen J., Mononen I. (2000). Enzyme replacement therapy in a mouse model of aspartylglycosaminuria. Faseb. J..

[29] Dunder U., Valtonen P., Kelo E., Mononen I. (2010). Early initiation of enzyme replacement therapy improves metabolic correction in the brain tissue of aspartylglycosaminuria mice. J. Inherit. Metab. Dis..

[30] Lin S.P., Chang J.H., de la Cadena M.P., Chang T.F., Lee-Chen G.J. (2007). Mutation identification and characterization of a Taiwanese patient with fucosidosis. J. Hum. Genet..

[31] Willems P.J., Gatti R., Darby J.K., Romeo G., Durand P., Dumon J.E., O’Brien J.S. (1991). Fucosidosis revisited: A review of 77 patients. Am. J. Med. Genet..

[32] Jiang M., Liu S., Jiang H., Lin Y., Shao Y., Hu H., Zhao X., Liu H., Huang Y., Liu L. (2017). Brain abnormalities in fucosidosis: Transplantation or supportive therapy?. Metab. Brain. Dis..

[33] Miano M., Lanino E., Gatti R., Morreale G., Fondelli P., Celle M.E., Stroppiano M., Crescenzi F., Dini G. (2001). Four year follow-up of a case of fucosidosis treated with unrelated donor bone marrow transplantation. Bone Marrow Transplant..

[34] Taylor R.M., Farrow B.R., Stewart G.J., Healy P.J., Tiver K. (1988). The clinical effects of lysosomal enzyme replacement by bone marrow transplantation after total lymphoid irradiation on neurologic disease in fucosidase deficient dogs. Transpl. Proc..

[35] Taylor R.M., Farrow B.R., Stewart G.J. (1992). Amelioration of clinical disease following bone marrow transplantation in fucosidase-deficient dogs. Am. J. Med. Genet..

[36] Vellodi A., Cragg H., Winchester B., Young E., Young J., Downie C.J., Hoare R.D., Stocks R., Banerjee G.K. (1995). Allogeneic bone marrow transplantation for fucosidosis. Bone Marrow Transplant..

[37] Gupta A., Lund T.C., Anderson N., Miettunen K., Eisengart J., Braunlin E., Orchard P.J. (2019). Allogeneic hematopoietic stem transplant improves outcome in fucosidosis. Mol. Genet. Metab.

[38] Kondagari G.S., Fletcher J.L., Cruz R., Williamson P., Hopwood J.J., Taylor R.M. (2015). The effects of intracisternal enzyme replacement versus sham treatment on central neuropathology in preclinical canine fucosidosis. Orphanet J. Rare Dis..

[39] Schiff M., Maire I., Bertrand Y., Cochat P., Guffon N. (2005). Long-term follow-up of metachronous marrow-kidney transplantation in severe type II sialidosis: What does success mean?. Nephrol. Dial. Transplant. Off. Publ. Eur. Dial. Transpl. Assoc. Eur. Ren. Assoc..

[40] Caciotti A., Melani F., Tonin R., Cellai L., Catarzi S., Procopio E., Chilleri C., Mavridou I., Michelakakis H., Fioravanti A. (2020). Type I sialidosis, a normosomatic lysosomal disease, in the differential diagnosis of late-onset ataxia and myoclonus: An overview. Mol. Genet. Metab..

[41] Lowden J.A., O’Brien J.S. (1979). Sialidosis: A review of human neuraminidase deficiency. Am. J. Hum. Genet..

[42] Ohshima T., Schiffmann R., Murray G.J., Kopp J., Quirk J.M., Stahl S., Chan C.C., Zerfas P., Tao-Cheng J.H., Ward J.M. (1999). Aging accentuates and bone marrow transplantation ameliorates metabolic defects in Fabry disease mice. Proc. Natl. Acad. Sci. USA.

[43] Wang D., Bonten E.J., Yogalingam G., Mann L., d’Azzo A. (2005). Short-term, high dose enzyme replacement therapy in sialidosis mice. Mol. Genet. Metab..

[44] Wenger D.A., Sujansky E., Fennessey P.V., Thompson J.N. (1986). Human beta-mannosidase deficiency. N. Engl. J. Med..

[45] Bedilu R., Nummy K.A., Cooper A., Wevers R., Smeitink J., Kleijer W.J., Friderici K.H. (2002). Variable clinical presentation of lysosomal beta-mannosidosis in patients with null mutations. Mol. Genet. Metab..

[46] Lund T.C., Miller W.P., Eisengart J.B., Simmons K., Pollard L., Renaud D.L., Wenger D.A., Patterson M.C., Orchard P.J. (2019). Biochemical and clinical response after umbilical cord blood transplant in a boy with early childhood-onset beta-mannosidosis. Mol. Genet. Genom. Med..

[47] Lund T.C., Cathey S.S., Miller W.P., Eapen M., Andreansky M., Dvorak C.C., Davis J.H., Dalal J.D., Devine S.M., Eames G.M. (2014). Outcomes after hematopoietic stem cell transplantation for children with I-cell disease. Biol. Blood Marrow Transplant. J. Am. Soc. Blood Marrow Transplant..

[48] Petrey A.C., Flanagan-Steet H., Johnson S., Fan X., De la Rosa M., Haskins M.E., Nairn A.V., Moremen K.W., Steet R. (2012). Excessive activity of cathepsin K is associated with cartilage defects in a zebrafish model of mucolipidosis II. Dis. Model. Mech..

[49] Flanagan-Steet H., Christian C., Lu P.N., Aarnio-Peterson M., Sanman L., Archer-Hartmann S., Azadi P., Bogyo M., Steet R.A. (2018). TGF-ss Regulates Cathepsin Activation during Normal and Pathogenic Development. Cell Rep..

[50] Wenger D.A., Sattler M., Clark C., Wharton C. (1976). I-cell disease: Activities of lysosomal enzymes toward natural and synthetic substrates. Life Sci..

[51] Leroy J.G., Spranger J.W., Feingold M., Opitz J.M., Crocker A.C. (1971). I-cell disease: A clinical picture. J. Pediatr..

[52] Cathey S.S., Kudo M., Tiede S., Raas-Rothschild A., Braulke T., Beck M., Taylor H.A., Canfield W.M., Leroy J.G., Neufeld E.F. (2008). Molecular order in mucolipidosis II and III nomenclature. Am. J. Med. Genetics. Part. A.

[53] Raas-Rothschild A., Bargal R., Goldman O., Ben-Asher E., Groener J.E., Toutain A., Stemmer E., Ben-Neriah Z., Flusser H., Beemer F.A. (2004). Genomic organisation of the UDP-N-acetylglucosamine-1-phosphotransferase gamma subunit (GNPTAG) and its mutations in mucolipidosis III. J. Med. Genet..

[54] Raas-Rothschild A., Cormier-Daire V., Bao M., Genin E., Salomon R., Brewer K., Zeigler M., Mandel H., Toth S., Roe B. (2000). Molecular basis of variant pseudo-hurler polydystrophy (mucolipidosis IIIC). J. Clin. Investig..

[55] Kudo M., Bao M., D’Souza A., Ying F., Pan H., Roe B.A., Canfield W.M. (2005). The alpha- and beta-subunits of the human UDP-N-acetylglucosamine:lysosomal enzyme N-acetylglucosamine-1-phosphotransferase [corrected] are encoded by a single cDNA. J. Biol. Chem..

[56] Oussoren E., van Eerd D., Murphy E., Lachmann R., van der Meijden J.C., Hoefsloot L.H., Verdijk R., Ruijter G.J.G., Maas M., Hollak C.E.M. (2018). Mucolipidosis type III, a series of adult patients. J. Inherit. Metab. Dis..

[57] Galjart N.J., Gillemans N., Harris A., van der Horst G.T., Verheijen F.W., Galjaard H., d’Azzo A. (1988). Expression of cDNA encoding the human "protective protein" associated with lysosomal beta-galactosidase and neuraminidase: Homology to yeast proteases. Cell.

[58] D’Azzo A., Hoogeveen A., Reuser A.J., Robinson D., Galjaard H. (1982). Molecular defect in combined beta-galactosidase and neuraminidase deficiency in man. Proc. Natl. Acad. Sci. USA.

[59] d’Azzo A., Andria G., Strisciuglio P., Galjaard H., Scriver C., Beaudet A., Sly W., Valle D. (2001). Galactosialidosis. The Metabolic and Molecular Bases of Inherited Disease.

[60] Zammarchi E., Donati M.A., Morrone A., Donzelli G.P., Zhou X.Y., d’Azzo A. (1996). Early-infantile galactosialidosis: Clinical, biochemical, and molecular observations in a new patient. Am. J. Med. Genet..

[61] Annunziata I., d’Azzo A. (2017). Galactosialidosis: Historic aspects and overview of investigated and emerging treatment options. Expert Opin. Orphan Drugs.

[62] Zhou X.Y., Morreau H., Rottier R., Davis D., Bonten E., Gillemans N., Wenger D., Grosveld F.G., Doherty P., Suzuki K. (1995). Mouse model for the lysosomal disorder galactosialidosis and correction of the phenotype with overexpressing erythroid precursor cells. Genes Dev..

[63] Hahn C.N., del Pilar Martin M., Zhou X.Y., Mann L.W., d’Azzo A. (1998). Correction of murine galactosialidosis by bone marrow-derived macrophages overexpressing human protective protein/cathepsin A under control of the colony-stimulating factor-1 receptor promoter. Proc. Natl. Acad. Sci. USA.

[64] Leimig T., Mann L., Martin Mdel P., Bonten E., Persons D., Knowles J., Allay J.A., Cunningham J., Nienhuis A.W., Smeyne R. (2002). Functional amelioration of murine galactosialidosis by genetically modified bone marrow hematopoietic progenitor cells. Blood.

[65] Bonten E.J., Wang D., Toy J.N., Mann L., Mignardot A., Yogalingam G., D’Azzo A. (2004). Targeting macrophages with baculovirus-produced lysosomal enzymes: Implications for enzyme replacement therapy of the glycoprotein storage disorder galactosialidosis. Faseb. J..

[66] Keulemans J.L., Reuser A.J., Kroos M.A., Willemsen R., Hermans M.M., van den Ouweland A.M., de Jong J.G., Wevers R.A., Renier W.O., Schindler D. (1996). Human alpha-N-acetylgalactosaminidase (alpha-NAGA) deficiency: New mutations and the paradox between genotype and phenotype. J. Med. Genet..

[67] van Diggelen O.P., Schindler D., Kleijer W.J., Huijmans J.M., Galjaard H., Linden H.U., Peter-Katalinic J., Egge H., Dabrowski U., Cantz M. (1987). Lysosomal alpha-N-acetylgalactosaminidase deficiency: A new inherited metabolic disease. Lancet.

[68] Kanekura T., Sakuraba H., Matsuzawa F., Aikawa S., Doi H., Hirabayashi Y., Yoshii N., Fukushige T., Kanzaki T. (2005). Three dimensional structural studies of alpha-N-acetylgalactosaminidase (alpha-NAGA) in alpha-NAGA deficiency (Kanzaki disease): Different gene mutations cause peculiar structural changes in alpha-NAGAs resulting in different substrate specificities and clinical phenotypes. J. Derm. Sci.

[69] Wang A.M., Schindler D., Desnick R. (1990). Schindler disease: The molecular lesion in the alpha-N-acetylgalactosaminidase gene that causes an infantile neuroaxonal dystrophy. J. Clin. Investig..

[70] Kodama K., Kobayashi H., Abe R., Ohkawara A., Yoshii N., Yotsumoto S., Fukushige T., Nagatsuka Y., Hirabayashi Y., Kanzaki T. (2001). A new case of alpha-N-acetylgalactosaminidase deficiency with angiokeratoma corporis diffusum, with Ménière’s syndrome and without mental retardation. Br. J. Derm..

[71] Fratantoni J.C., Hall C.W., Neufeld E.F. (1968). Hurler and Hunter syndromes: Mutual correction of the defect in cultured fibroblasts. Science.

[72] Neufeld E.F. (2011). From serendipity to therapy. Annu Rev. Biochem.

[73] Krivit W., Pierpont M.E., Ayaz K., Tsai M., Ramsay N.K., Kersey J.H., Weisdorf S., Sibley R., Snover D., McGovern M.M. (1984). Bone-marrow transplantation in the Maroteaux-Lamy syndrome (mucopolysaccharidosis type VI). Biochemical and clinical status 24 months after transplantation. N. Engl. J. Med..

[74] Hobbs J.R., Hugh-Jones K., Barrett A.J., Byrom N., Chambers D., Henry K., James D.C., Lucas C.F., Rogers T.R., Benson P.F. (1981). Reversal of clinical features of Hurler’s disease and biochemical improvement after treatment by bone-marrow transplantation. Lancet.

[75] Capotondo A., Milazzo R., Politi L.S., Quattrini A., Palini A., Plati T., Merella S., Nonis A., di Serio C., Montini E. (2012). Brain conditioning is instrumental for successful microglia reconstitution following hematopoietic stem cell transplantation. Proc. Natl. Acad. Sci. USA.

[76] Biffi A., Capotondo A., Fasano S., del Carro U., Marchesini S., Azuma H., Malaguti M.C., Amadio S., Brambilla R., Grompe M. (2006). Gene therapy of metachromatic leukodystrophy reverses neurological damage and deficits in mice. J. Clin. Investig..

[77] Biffi A., De Palma M., Quattrini A., Del Carro U., Amadio S., Visigalli I., Sessa M., Fasano S., Brambilla R., Marchesini S. (2004). Correction of metachromatic leukodystrophy in the mouse model by transplantation of genetically modified hematopoietic stem cells. J. Clin. Investig..

[78] Wilkinson F.L., Sergijenko A., Langford-Smith K.J., Malinowska M., Wynn R.F., Bigger B.W. (2013). Busulfan conditioning enhances engraftment of hematopoietic donor-derived cells in the brain compared with irradiation. Mol. Ther. J. Am. Soc. Gene Ther..

